# Exercise-induced brachial–ankle pulse wave velocity changes before and after transcatheter aortic valve replacement

**DOI:** 10.1007/s00380-025-02593-9

**Published:** 2025-08-18

**Authors:** Ayano Osawa, Hiroki Ikenaga, Atsushi Kuraishi, Kiyotaka Togi, Mikio Shigehara, Ayano Hamada, Makoto Takeuchi, Yohei Hyodo, Atsuo Mogami, Akane Tsuchiya, Atsushi Takeda, Takayuki Nakano, Yusuke Ueda, Kosuke Takahari, Yuichi Morita, Tasuku Higashihara, Noriaki Watanabe, Yoshiharu Sada, Hiroto Utsunomiya, Taiichi Takasaki, Shinya Takahashi, Yukiko Nakano

**Affiliations:** 1 Department of Cardiovascular Medicine, Hiroshima University Graduate School of Biomedical and Health Sciences, 1-2-3 Kasumi, Hiroshima, Minami-ku 734-8551 Japan; 2 Department of Cardiovascular Medicine, Hiroshima City Hiroshima Citizens Hospital, Hiroshima, Japan; 3 Department of Surgery, Hiroshima University Graduate School of Biomedical and Health Sciences, Hiroshima, Japan

**Keywords:** Aortic stenosis, Transcatheter aortic valve replacement, Brachial–ankle pulse wave velocity, Arterial stiffness

## Abstract

Elevated arterial stiffness is associated with cardiovascular risk. Brachial–ankle pulse wave velocity (baPWV), a measure of arterial stiffness, is decreased by exercise stress, which is associated with good vascular endothelial function. Moreover, baPWV may predict outcomes following transcatheter aortic valve replacement (TAVR) and has been reported to change before and after TAVR. However, studies on baPWV changes in patients with TAVR undergoing exercise stress have not been conducted. This study aimed to assess the changes in baPWV before and after TAVR using a simple exercise stress method. We enrolled 40 patients (mean age, 84.6 ± 4.4 years; 45% males) with severe symptomatic aortic stenosis undergoing TAVR. baPWV was assessed at rest and immediately following the exercise protocol. Exercise stress was performed using a simple method wherein patients actively plantar flexed and dorsiflexed their legs in a resting supine position. Measurements were conducted at baseline and after TAVR. Resting baPWV significantly increased from 1673 ± 322 to 2073 ± 426 cm/s (*p* < 0.001), and exercise stress baPWV also significantly increased from 1662 ± 339 to 1972 ± 335 cm/s (*p* < 0.001) after TAVR. Compared with resting baPWV, post-exercise baPWV did not change before TAVR (from 1673 ± 322 to 1662 ± 339 cm/s, *p* = 0.68), but significantly decreased after TAVR (from 2073 ± 426 to 1972 ± 335 cm/s, *p* = 0.012). The arterial system demonstrated increased baPWV in response to the acute relief of the obstruction following TAVR. Exercise stress decreased baPWV following TAVR, suggesting that endothelial function was maintained, which was masked before TAVR.

## Introduction

Aortic stenosis (AS), a gradually progressive disease, is characterized by calcified narrowing of the aortic valve, leading to sudden death and cardiovascular complications. It affects approximately 2%–7% of the population aged >65 years, and its prevalence is anticipated to dramatically increase over the next few decades [1–4]. Transcatheter aortic valve replacement (TAVR) offers a groundbreaking treatment option for severe AS, rivaling the gold standard surgical aortic valve replacement [1, 2]. Most of the patients with AS are older adults and at high surgical risk; fortunately, TAVR is widely available to such high-risk patients and is globally recognized and rapidly expanding [5].

Arterial dysfunction is characterized by arterial wall thickening, impaired endothelial and autonomic function, and increased arterial stiffness [6, 7]. Brachial–ankle pulse wave velocity (baPWV) is widely accepted as an arterial stiffness indicator. Increased resting PWV is associated with the risk of cardiovascular events and mortality [8, 9]. Conversely, long-term exercise causes improved PWV [10, 11]. Among healthy participants, PWV acutely decreases during a hyperemia-induced increase in flow, but not when flow-related endothelium-mediated vasodilation is impaired [12, 13]. In other words, the exercise stress-induced decrease in PWV suggests the preservation of good vascular endothelial function [14]. Several studies have reported the following associations between resting PWV and prognosis in patients undergoing TAVR: the association between the rate of change in baPWV before and after TAVR and all-cause mortality [15], the association between PWV values before TAVR and the composite event of all-cause mortality and heart failure-related rehospitalization [16], and the association between invasively measured PWV intraoperatively and all-cause mortality [17]. Moreover, PWV is reported to change following TAVR [18]. However, the exercise-induced dynamics of PWV following TAVR remain inadequately investigated. Therefore, this study aimed to investigate the immediate effects of exercise stress on baPWV in patients undergoing TAVR.

## Materials and methods

### Study population

**Fig. 1:**
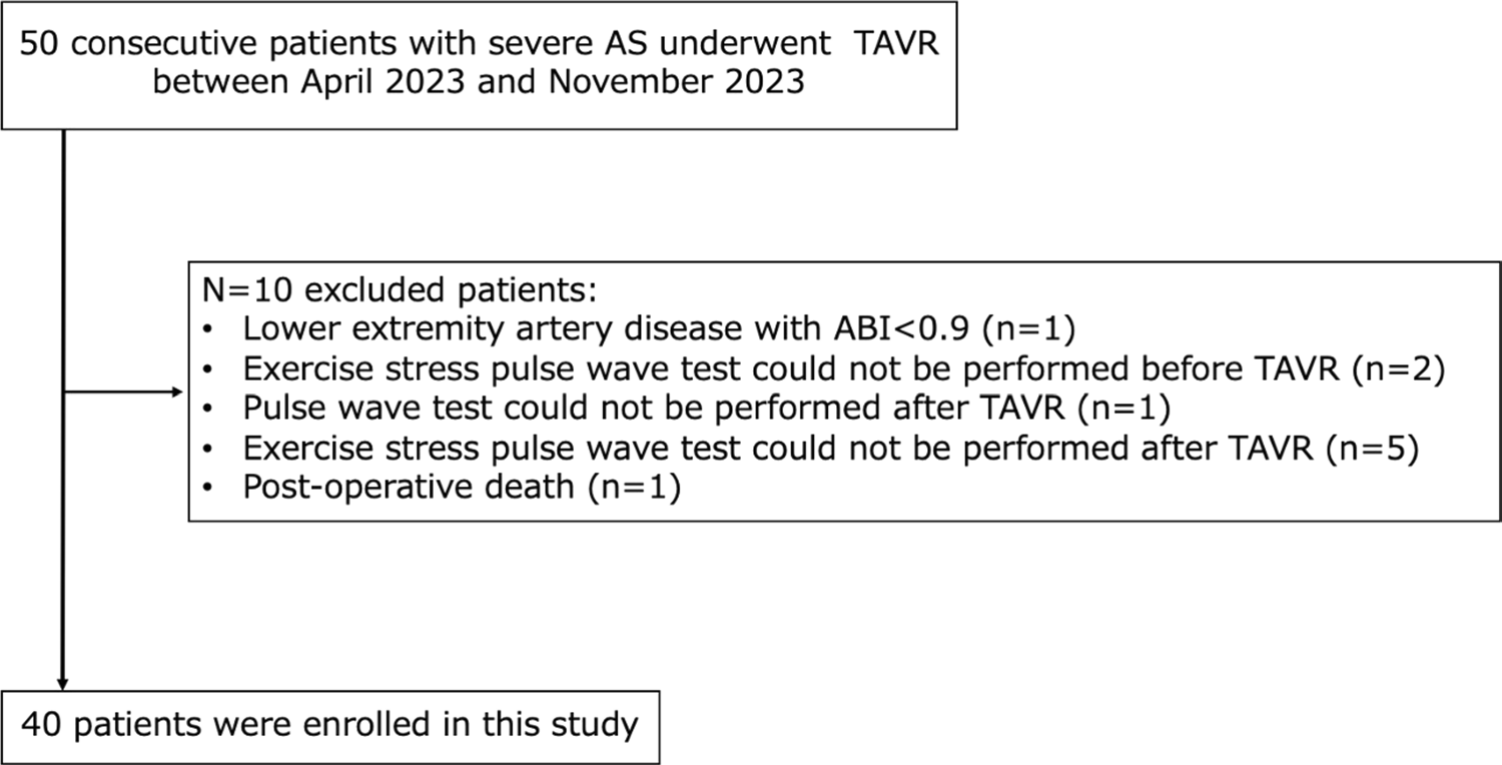
Patient selection flowchart and workflow. *ABI*, ankle–brachial index; *AS*, aortic stenosis; *baPWV*, brachial–ankle pulse wave velocity; *TAVR*, transcatheter aortic valve replacement

We prospectively enrolled 50 consecutive patients with symptomatic severe AS who underwent TAVR at our hospital between April 2023 and November 2023. Severe AS was defined as an aortic valve area (AVA) of <1.0 cm^2^ using the continuity equation (or AVA index (AVAi) < 0.6 cm^2^/m^2^), a mean gradient of >40 mmHg, or an aortic valve (AV) peak velocity of >4.0 m/s using resting echocardiography. The peak AV velocity following a low-dose dobutamine stress test was defined as >4.0 m/s when the left ventricular ejection fraction (LVEF) was reduced (LVEF < 50%). The following patients were excluded: one patient who did not undergo baPWV test after TAVR; one patient with arteriosclerosis obliterans, defined by an ankle–brachial index (ABI) value of <0.9; seven patients who did not undergo stress test before or after TAVR; and one patient who died upon discharge, resulting in a total of 40 patients (Fig. 1). This study complied with the Declaration of Helsinki. The research ethics committee of our institution approved the study protocol. All patients provided opt-out consent.

### TAVR procedure

The patient’s indication for TAVR and the treatment strategy are identified by a heart team comprising cardiologists, cardiac surgeons, anesthesiologists, and imaging specialists. All patients underwent TAVR using standard procedures. All interventions were performed in a hybrid operating room under general anesthesia, using balloon-expandable or self-expandable valves. Hemodynamic parameter measurements were performed at baseline (day before TAVR–25 days) and at discharge (4–14 days following TAVR based on the duration of the patient’s hospital stay) in a dedicated laboratory with good reproducibility of measurements. All data on each patient’s medical history and medication status were collected and input into a dedicated database.

### baPWV measurement method

baPWV was noninvasively measured using a validated oscillometric device (BP-203RPE III; Omron Colin, Tokyo, Japan), with pressure cuffs wrapped on each brachial arm and ankle [19–22]. Furthermore, ABI values were simultaneously measured using this device. Patients were examined after at least a 5-min rest in the supine position. After baPWV was measured at rest, the exercise stress was performed in the resting supine position. Patients performed the exercise stress by a simple method of active plantar flexion and dorsiflexion of the legs while in the resting supine position with the knee fully extended. The speed was performed at the maximum tolerable speed (at least one flexion or dorsiflexion per second). The duration was limited to a maximum of 1 min, and if the session could not be continued for the full 1 min, the session was interrupted and baPWV was assessed. The reason for the 1-min limit was that several patients perceived that continuing the exercise beyond 1 min would be difficult. Peripheral blood pressure (BP) in both upper arms was measured using an automated oscillometric device (BP-203RPE III; Omron Colin, Tokyo, Japan), which was measured simultaneously with baPWV at rest and during exercise stress. The average BP for each of the left and right arms was used. The heart rate (HR) was also measured simultaneously with baPWV at rest and during exercise stress.

### Laboratory data

Laboratory data were measured on admission. The estimated glomerular filtration rate (eGFR) was calculated using the following Japanese formula: eGFR (mL/min/1.73 m^2^) = 194 × age − 0.287 × Cre − 1.094 (× 0.739 for females) [23].

### Echocardiographic assessment

An experienced sonographer performed transthoracic echocardiography before TAVR and at 2 weeks following TAVR using commercially available ultrasonographic equipment (Vivid E95, Vivid E9; GE Vingmed Ultrasound, Horten, Norway; EPIQ, Affinity; Philips Ultrasound, Bothell, WA, USA; Artida2; Canon Medical Systems, Tochigi, Japan). Echocardiographic measurements were performed according to the recommendations of the American Society of Echocardiography [24]. The AV mean gradient and peak velocity of the transaortic valve flow were measured from the apical view. Using a two-dimensional Doppler method with continuous equations, the AVA was estimated. The LVEF was measured using the biplane-modified Simpson method from the apical view.

## Statistical analysis

Continuous variables were expressed as means ± standard deviations and medians (interquartile ranges) for normally and nonnormally distributed data, respectively. Student’s t test or the nonparametric Wilcoxon signed rank test was used for comparison between the two groups for continuous variables. Categorical variables were presented as numbers and percentages. Using the chi-square test, group differences were evaluated for categorical variables. baPWV and BP values were averaged for the left and right arms. Univariate analysis of variance was used for assessing the changes in the population from the baseline to post-TAVR. Pearson’s correlation coefficient was used for examining the correlations between study variables. Univariate logistic regression analyses were used to identify independent predictor of increased baPWV or decreased baPWV after TAVR. *P* value <0.05 was considered statistically significant. Statistical analyses were performed using JMP 17 (SAS Institute Inc., Cary, NC, USA).

## Results

### Baseline patient characteristics

The baseline characteristics of the study participants are presented in Table 1. Their mean age was 84.6 ± 4.4 years, and 45% of them were male. Hypertension (77.5%) and dyslipidemia (47.5%) constituted a substantial proportion, reflecting common comorbidities. Additionally, 27.5% and 17.5% of the patients had diabetes and coronary artery disease, respectively. Of the patients, 27.5% had New York Heart Association functional class III or IV, and the median N-terminal pro-brain natriuretic peptide (NTproBNP) level was 1028 pg/mL. The majority of patients were treated with antihypertensive drugs (angiotensin-converting enzyme inhibitors/angiotensin receptor blockers/angiotensin receptor-neprilysin inhibitors [77.5%] and calcium channel blockers [40%]) and diuretics (52.5%). The mean AV peak velocity, AVA, peak pressure gradient, and mean pressure gradient were 4.5 ± 0.6 m/s, 0.74 ± 0.19 cm^2^, 79.2 ± 23.3 mmHg, and 45.7 ± 17.2 mmHg, respectively. Additionally, the LVEF was preserved with a mean of 60% ± 12%.

**Table 1:** Baseline characteristics of the study participants

	Total population (*N* = 40)
Age (years)	84.6 ± 4.4
Gender (male/female)	18 (45%)/22 (55%)
Height (cm)	151.4 ± 10.0
Wight (kg)	53.3 ± 10.5
Body mass index (kg/m^2^)	23.4 ± 5.2
Body surface area (m^2^)	1.48 ± 0.16
*Cardiovascular risk factors*	
Hypertension, n (%)	31 (77.5)
Dyslipidemia, n (%)	19 (47.5)
Diabetes mellitus, n (%)	11 (27.5)
Current smoker, n (%)	2 (5)
Coronary artery disease, n (%)	7 (17.5)
Previous PCI, n (%)	5 (12.5)
Previous CABG, n (%)	1 (2.5)
History of heart surgery, n (%)	1 (2.5)
Cerebrovascular disease, n (%)	4 (10)
Peripheral artery disease, n (%)	2 (5)
NYHA III/IV, n (%)	11 (27.5)
Atrial fibrillation, n (%)	7 (17.5)
Permanent pacemaker at baseline, n (%)	3 (7.5)
Chronic obstructive pulmonary disease, n (%)	2 (5)
Chronic kidney disease, n (%)	4 (10)
Hemodialysis, n (%)	0 (0)
6 min walk distance (m)	332.0 ± 128.5
*Medications*	
DOAC, n (%)	7 (17.5)
Aspirin, n (%)	5 (12.5)
Statins, n (%)	18 (45)
Ezetimibe, n (%)	3 (7.5)
Calcium channel blockers, n (%)	16 (40)
ACE-I/ARB/ARNi, n (%)	31 (77.5)
β-Blockers, n (%)	8 (20)
Diuretics, n (%)	21 (52.5)
*Laboratory data*	
eGFR (mL/min/1.73m^2^)	50.0 ± 18.1
NTproBNP (pg/mL)	1028 (324−1772)
*Echocardiographic indices*	
AV peak velocity (m/s)	4.5 ± 0.6
AVA (cm^2^)	0.74 ± 0.19
AVAi (cm^2^/m^2^)	0.51 ± 0.15
Peak pressure gradient (mmHg)	79.2 ± 23.3
Mean pressure gradient (mmHg)	45.7 ± 17.2
Left ventricular ejection fraction (%)	60 ± 12
LVDd (mm)	46.0 ± 7.4
LVDs (mm)	32.0 ± 8.4
IVST (mm)	10.1 ± 2.2
PWT (mm)	10.1 ± 2.1
AR ≧moderate, n (%)	1 (2.5)
MR ≧moderate, n (%)	1 (2.5)

Data are expressed as the number of patients (percentage), means ± standard deviations (SD) and medians (interquartile ranges)

*ACE-I* angiotensin-converting enzyme inhibitors; *AR* aortic regurgitation; *ARB* angiotensin receptor blockers; *ARNi* angiotensin receptor-neprilysin inhibitors; *AV* aortic valve; *AVA* aortic valve area; *AVAi* aortic valve area index; *CABG* coronary artery bypass graft; *DOAC* Direct oral anticoagulants; *eGFR* estimated Glomerular Filtration Rate; *IVST* interventricular septum thickness; *LVDd* left ventricle diastolic diameter; *LVDs* left ventricle systolic diameter; *MR* mitral regurgitation; *NT-proBNP* N-terminal pro-brain natriuretic peptids; *NYHA* New York Heart Association; *PCI* percutaneous coronary intervention; *PWT* posterior wall thickness

### Changes before and after TAVR

**Fig. 2:**
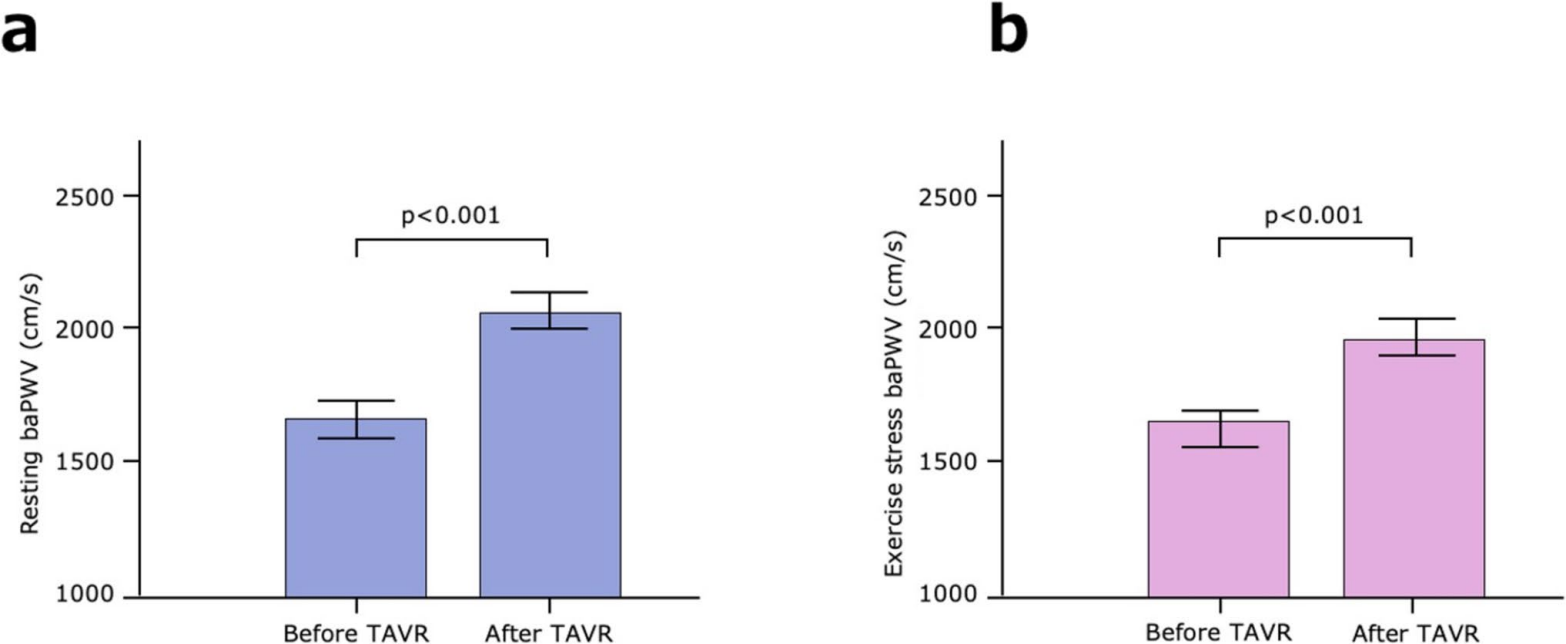
Changes in baPWV before and after TAVR. (**a**) Changes in resting baPWV before and after TAVR. Resting baPWV is significantly increased after TAVR (from 1673 ± 322 to 2073 ± 426 cm/s, *p* < 0.001). (**b**) Changes in exercise stress baPWV before and after TAVR. baPWV at exercise stress is also significantly increased after TAVR (from 1662 ± 339 to 1972 ± 335 cm/s, *p* < 0.001). P values by analysis of variance (ANOVA). The range of bars on the graph indicates the standard error (SE). *baPWV*, brachial–ankle pulse wave velocity; *TAVR*, transcatheter aortic valve replacement

The changes in the hemodynamic parameters and echocardiographic indices before and after TAVR are shown in Table 2. At baseline, the mean resting baPWV was 1673 ± 322 cm/s, and the mean exercise stress baPWV was 1662 ± 339 cm/s. The AV peak velocity and mean pressure gradient were significantly decreased and the AVA was significant increased before and after TAVR. These findings indicate the improvement of AS using TAVR. Following TAVR, resting baPWV was significantly increased (from 1673 ± 322 to 2073 ± 426 cm/s, *p* < 0.001, Fig. 2a). Similarly, following TAVR, exercise stress baPWV was significantly increased (from 1662 ± 339 to 1972 ± 335 cm/s, *p* < 0.001, Fig. 2b). Before and after TAVR, no significant changes in LVEF were observed (from 59% ± 12% to 61% ± 9%, *p* = 0.197). No significant differences in the change in BP at rest after TAVR were noted (from 131.7 ± 22.4 to 134.8 ± 16.6 mmHg, *p* = 0.40 and from 64.7 ± 9.5 to 64.2 ± 8.4 mmHg, *p* = 0.81 for systolic and diastolic BP, respectively). No significant differences in the change in the HR before and after TAVR were noted (from 66.9 ± 11.0 to 69.2 ± 12.2 beats/min [bpm], *p* = 0.163). At rest, no significant differences in the change of BP and HR at exercise stress before and after TAVR were observed (from 140.0 ± 22.6 to 138.7 ± 15.7 mmHg, *p* = 0.68 and from 67.8 ± 9.6 to 65.8 ± 9.5 mmHg, *p* = 0.32 for systolic and diastolic BP, respectively, and from 68.4 ± 11.8 to 69.4 ± 12.4 bpm, *p* = 0.57 for HR).

**Table 2:** Changes in the hemodynamic parameters and echocardiographic indices before and after TAVR

	Before TAVR (*n* = 40)	After TAVR (*n* = 40)	*P* value
*Hemodynamic parameters*			
Rest			
Peripheral systolic blood pressure (mmHg)	131.7 ± 22.4	134.8 ± 16.6	0.40
Peripheral diastolic blood pressure (mmHg)	64.7 ± 9.5	64.2 ± 8.4	0.81
Heart rate (beats/min [bpm])	66.9 ± 11.0	69.2 ± 12.2	0.163
Resting baPWV (cm/s)	1673 ± 322	2073 ± 426	<0.001
Exercise stress			
Peripheral systolic blood pressure (mmHg)	140.0 ± 22.6	138.7 ± 15.7	0.68
Peripheral diastolic blood pressure (mmHg)	67.8 ± 9.6	65.8 ± 9.5	0.32
Heart rate (beats/min [bpm])	68.4 ± 11.8	69.4 ± 12.4	0.57
Exercise stress baPWV (cm/s)	1662 ± 339	1972 ± 335	<0.001
*Echocardiographic indices*			
AV peak velocity (m/s)	4.4 ± 0.6	2.2 ± 0.5	<0.001
AVA (cm^2^)	0.74 ± 0.19	1.58 ± 0.34	<0.001
AVAi (cm^2^/m^2^)	0.51 ± 0.15	1.08 ± 0.26	<0.001
Peak pressure gradient (mmHg)	79.2 ± 23.3	20.8 ± 9.4	<0.001
Mean pressure gradient (mmHg)	45.7 ± 17.2	11.0 ± 5.1	<0.001
Left ventricular ejection fraction (%)	59 ± 12	61 ± 9	0.197

Continuous variables with normal distribution are presented as means ± standard deviations (SD)

*AV* aortic valve; *AVA* aortic valve area; *AVAi* aortic valve area index; *baPWV* brachial–ankle pulse wave velocity; *TAVR* transcatheter aortic valve replacement

### Changes in exercise stress

**Fig. 3:**
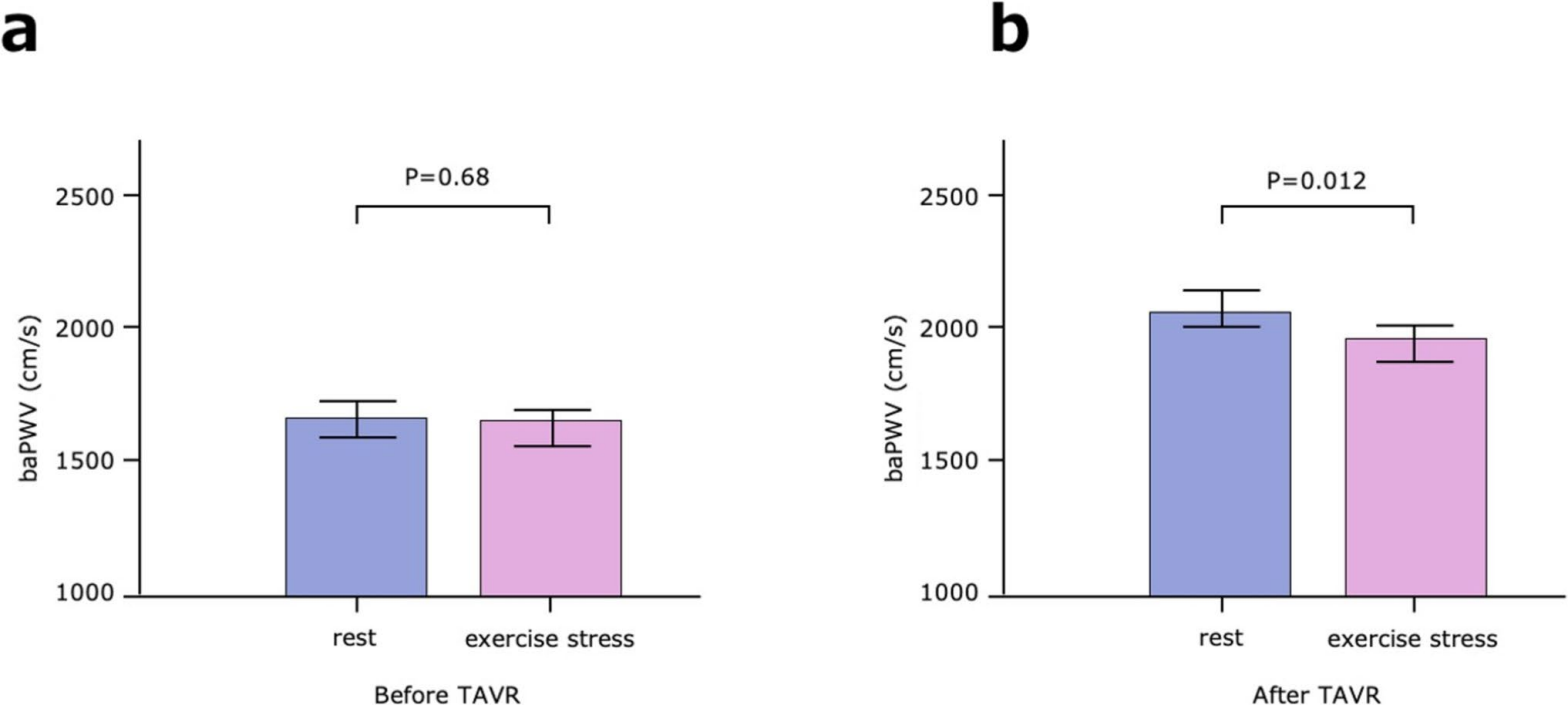
Changes in baPWV with exercise stress before and after TAVR. (**a**) Changes in baPWV with exercise stress before TAVR. Before TAVR, no significant differences in the changes in baPWV from rest to post-exercise stress are noted (from 1673 ± 322 to 1662 ± 339 cm/s, *p* = 0.68). (**b**) Changes in baPWV with exercise stress after TAVR. After TAVR, baPWV is significantly decreased from rest to exercise stress (from 2073 ± 426 to 1972 ± 335 cm/s, *p* = 0.012). *P* values by ANOVA. The range of bars on the graph indicates the SE. *baPWV*, brachial–ankle pulse wave velocity; *TAVR*, transcatheter aortic valve replacement

Before TAVR, no significant differences in the change in baPWV from rest to post-exercise stress were noted (from 1673 ± 322 to 1662 ± 339 cm/s, *p* = 0.68, Fig. 3a). However, following TAVR, baPWV was significantly decreased from rest to post-exercise stress (from 2073 ± 426 to 1972 ± 335 cm/s, *p* = 0.012, Fig. 3b).

Univariate logistic regression analysis to identify the association between changes of AS improvement after TAVR [ΔAV peak velocity (m/s), ΔAVA (cm2), ΔAVAi (cm2/m2), ΔAV peak pressure gradient (mmHg), ΔAV mean pressure gradient (mmHg) and ΔLVEF (%)] and increased baPWV after TAVR was shown in Table 3. There were no significant correlation between the increase in baPWV after TAVR and the degree of improvement of AS, except between ΔAV peak velocity and the increase in exercise stress baPWV (odds ratio [OR] 1.17, 95% confidence interval [CI] 1.01–1.52, *p* = 0.03). In addition, univariate logistic regression analysis to identify the association between decreased exercise stress baPWV after TAVR and changes of clinical biomarkers, echocardiographic indices are shown in Table 4. There was no significant correlation between decreased exercise stress baPWV after TAVR and ΔNTproBNP or Δechocardiographic parameters.

**Table 3:** Univariate logistic regression analysis of the association between increased baPWV after TAVR and changes of aortic stenosis improvement after TAVR

	OR	OR 95% CI	*P* value
For resting baPWV			
ΔAV peak velocity (per 1 m/s increase)	1.03	0.97–1.21	0.42
ΔAVA (per 1 cm^2^ increase)	0.77	0.45–1.20	0.26
ΔAVAi (per 1 cm^2^/m^2^ increase)	1.26	0.81–2.13	0.33
ΔPeak pressure gradient (per 1 mmHg increase)	0.99	0.95–1.01	0.38
ΔMean pressure gradient (per 1 mmHg increase)	1.00	0.99–1.01	0.59
ΔLeft ventricular ejection fraction (per 1 % increase)	1.04	0.93–1.20	0.46
For exercise stress baPWV			
ΔAV peak velocity (per 1 m/s increase)	1.17	1.01–1.52	0.03
ΔAVA (per 1 cm^2^ increase)	1.22	0.72–2.03	0.44
ΔAVAi (per 1 cm^2^/m^2^ increase)	0.79	0.46–1.34	0.37
ΔPeak pressure gradient (per 1 mmHg increase)	0.97	0.90–1.00	0.07
ΔMean pressure gradient (per 1 mmHg increase)	1.00	0.99–1.02	0.73
ΔLeft ventricular ejection fraction (per 1 % increase)	1.13	0.99–1.37	0.07

*OR* odds ratio; *CI* confidence interval; *baPWV* brachial–ankle pulse wave velocity; *TAVR* transcatheter aortic valve replacement; *AV* aortic valve; *AVA* aortic valve area; *AVAi* aortic valve area index

**Table 4:** Univariate logistic regression analysis of the association between changes of clinical biomarkers, echocardiographic indices and decreased in exercise stress baPWV after TAVR

	OR	OR 95% CI	*P* value
*Clinical biomarkers*			
ΔNTproBNP (per 1 pg/mL increase)	1.00	0.99-1.01	0.88
*Echocardiographic indices*			
ΔLeft ventricular ejection fraction (per 1 % increase)	1.01	0.91-1.12	0.83
ΔLAD (per 1 mm increase)	1.02	0.98-1.05	0.31
ΔLVDd (per 1 mm increase)	1.06	0.38-2.58	0.90
ΔLVDs (per 1 mm increase)	1.01	0.65-1.73	0.95
ΔIVST (per 1 mm increase)	1.03	0.92-1.14	0.65
ΔPWT (per 1 mm increase)	0.96	0.85-1.08	0.49
ΔStroke volume (per 1 mL increase)	1.01	0.79-1.31	0.96

*OR* odds ratio; *CI* confidence interval; *baPWV* brachial–ankle pulse wave velocity; *TAVR* transcatheter aortic valve replacement; *NT-proBNP* N-terminal pro-brain natriuretic peptids; *LAD* left atrial diameter; *LVDd* left ventricle diastolic diameter; *LVDs* left ventricle systolic diameter; *IVST* interventricular septum thickness; *PWT* posterior wall thickness

## Discussion

This study investigated the changes in baPWV at rest and after exercise stress in patients undergoing TAVR and the acute effects of exercise on baPWV. This study mainly revealed the following: (1) arterial stiffness assessed by baPWV increased both at rest and at exercise stress after TAVR. baPWV at rest was significantly increased after TAVR; similarly, baPWV at exercise stress after TAVR was significantly increased. (2) Before TAVR, baPWV did not change after exercise; however, after TAVR, baPWV showed a significant decrease after exercise. To the best of our knowledge, there have been no previous studies that have investigated the changes in baPWV with exercise stress before and after TAVR.

AS, a progressive condition, leads to sudden death and cardiovascular complications; however, the introduction of TAVR enables the minimally invasive treatment of AS [1, 2, 5]. Among patients with severe AS, various studies have been conducted to determine which patients may most likely benefit from TAVR and identify the prognostic indicators. Characterized by aortic valve calcification, this condition is predominantly attributed to atherosclerotic processes. The quantitative assessment of atherosclerosis and vascular function is essential in patients with severe AS to prevent cardiovascular complications. Surrogate markers, including baPWV, are used in clinical settings worldwide. Mitchell and Chirinos reported that increased resting PWV was associated with the risk of cardiovascular events and mortality [8, 9]. Moreover, several studies have suggested that resting PWV can be a prognostic factor even in patients undergoing TAVR. Tanaka et al. evaluated the baseline baPWV before TAVR and reported that high baPWV was an independent risk factor for the composite outcome of all-cause mortality and heart failure-related rehospitalization following TAVR [16]. Toma et al. evaluated the changes in baPWV rather than the baseline values and reported that an increase in baPWV was significantly associated with event-free survival following TAVR [15]. Broyd et al. simultaneously recorded invasive pressure data from the femoral head and aortic root before TAVR and calculated the wave time using an automated foot-to-foot methodology. Distance was measured from the pre-TAVR computed tomography, and PWV was calculated on the basis of these values. They demonstrated that invasive aortic PWV can be conveniently and accurately measured during TAVR and reported that intraoperative PWV was the only strong predictor of 1-year post-procedure mortality [17]. Although PWV may be a potential prognostic factor following TAVR, whether PWV should be used before or after TAVR and whether a single point value or change in PWV should be used remain inconsistent.

### Reasons for PWV optimization

baPWV is a measure of arterial stiffness that is significantly influenced by TAVR. The underlying mechanism of change following TAVR can be attributed to alterations in the hemodynamic conditions following the procedure. After TAVR, aortic valve obstruction is relieved, load transmission to the arterial system is enhanced, and to withstand this load, the aortic wall responds to acute pressure and volume changes not only by existing stiffness but also by passive stiffness that increases owing to long-term employed stiff wall components, including collagen fibers [25, 26]. Although the potential role of active responses, including increased smooth muscle cell tone, remains unclear, these changes after TAVR suggest that peripheral vasodilation is a compensatory mechanism for the hemodynamic changes [27]. Following relief of obstruction and restoration of cardiac output, peripheral resistance, which was increased in patients with AS to maintain peripheral tissue perfusion, is anticipated to decrease owing to peripheral vasodilation. However, these mechanisms remain speculative, and the mechanisms of these changes are not well elucidated.

### Reasons for post-exercise PWV optimization following TAVR

A better understanding of how these changes and responses are affected by the addition of exercise stress may offer enhanced understanding into this mechanism in patients undergoing TAVR. Compared with baseline levels, the post-exercise PWV has been reported to either increase [28, 29], decrease [30–32], or remain unchanged [33, 34]. This variability in the PWV response following exercise may be due to the anatomical segment being assessed, the frequency of measurements, and the age and health status of the study participants [35]. Katerina et al. demonstrated that PWV immediately after exercise was lower than the pre-exercise baseline levels in healthy participants [14]. Maria et al. showed that PWV decreased following exercise in healthy older adults [36]. This decrease in PWV is related to the peripheral vasodilation of the lower extremities immediately after exercise. In healthy participants, PWV has demonstrated sensitivity to acute changes in vascular tone, independent of changes in BP. The decreased PWV following exercise is associated with increased endothelial function via shear, activation of vasodilators including nitric oxide and prostaglandins, and suppression of vasoconstrictors including endothelin-1 expression [37–39]. In healthy participants, PWV acutely decreases during a hyperemia-induced increase in flow, but not when flow-related endothelium-mediated vasodilation is impaired [12, 13]. In other words, the exercise stress-induced decrease in PWV suggests the preservation of good vascular endothelial function [14]. We here observed that compared with resting baPWV, baPWV after exercise stress was unchanged before TAVR, whereas it significantly decreased following TAVR. This change suggests the maintenance of vascular endothelial function in patients with severe AS that was masked before TAVR. baPWV measures vascular stiffness, calculated as PWV = pulse wave velocity distance/pulse wave transit time. In severe AS, excessive peripheral vasoconstriction to compensate for reduced cardiac output prevents the normal decrease in PWV after exercise [15, 18]. After TAVR, the response resembles that of healthy individuals, suggesting that severe AS masks baPWV changes, likely due to vasoconstriction caused by decreased cardiac output. In our study, there was significant correlation between the increase in exercise stress baPWV after TAVR and ΔAV peak velocity. This may be one mechanism for the improvement of baPWV after TAVR. In patients with AS, pre-TAVR baPWV cannot be accurately assessed. Although baPWV has attracted attention as a prognostic factor following TAVR, which values to use before and after TAVR remains a matter for consideration.

## Limitations

This study had several limitations. First, it was a single-center retrospective observational study. Second, the data on baPWV were small, obtained from 40 participants, and should be interpreted with caution. Third, the application of baPWV in patients with severe AS remained unclear. Older adult patients with severe AS who have various comorbidities and are undergoing TAVR experience significant arterial stiffness, which may result in the inaccurate estimation of baPWV using oscillometric methods. However, several previous studies reported that comparing baPWV values among patients with severe AS is acceptable [18, 40, 41]. In patients with severe AS, baPWV may be underevaluated. In our study, we performed exercise stress baPWV before and after TAVR and observed the changes in baPWV, suggesting limitations in the accurate assessment of baPWV before TAVR. Fourth, exercise stress was the original method. No previous studies employed such a simple method of exercise stress for investigating the changes in baPWV; therefore, further research is warranted. However, as patients with severe AS are older and at high surgical risk, careful consultation is necessary to perform exercise stress on a treadmill or ergometer and compare the results. Finally, we did not measure potential mechanisms, including endothelial function, involved in exercise-induced changes in arterial stiffness. It remains unclear what factors improve exercise stress baPWV after TAVR, and this should be the subject of future research.

## Conclusions

Following TAVR, baPWV significantly increased both at rest and after exercise stress. Furthermore, exercise stress decreased baPWV following TAVR, suggesting that endothelial function was maintained, which was masked before TAVR. To the best of our knowledge, this is the first study to elucidate the changes in baPWV before and after TAVR caused by exercise stress. Further studies should be conducted to understand the mechanisms of baPWV changes with exercise stress and investigate the prognostic impact of baPWV changes with exercise stress in patients who underwent TAVR.

## Data Availability

The data used in this study cannot be shared publicly to protect the privacy of the individuals who participated in the study. The data will be shared upon reasonable request to the corresponding author.
